# Risk of Post-Discharge Venous Thromboembolism and Associated Mortality in General Surgery: A Population-Based Cohort Study Using Linked Hospital and Primary Care Data in England

**DOI:** 10.1371/journal.pone.0145759

**Published:** 2015-12-29

**Authors:** George Bouras, Elaine Marie Burns, Ann-Marie Howell, Alex Bottle, Thanos Athanasiou, Ara Darzi

**Affiliations:** 1 Department of Surgery and Cancer, Imperial College, St Mary’s Hospital, Praed Street, London, W21NY, United Kingdom; 2 Department of Epidemiology and Public Health, Imperial College, Charing Cross Hospital, 3 Dorset Rise, London, EC4Y 8EN, United Kingdom; Emory University School of Medicine, UNITED STATES

## Abstract

**Background:**

Trends towards day case surgery and enhanced recovery mean that postoperative venous thromboembolism (VTE) may increasingly arise after hospital discharge. However, hospital data alone are unable to capture adverse events that occur outside of the hospital setting. The National Institute for Health and Care Excellence has suggested the use of primary care data to quantify hospital care-related VTE. Data in surgical patients using these resources is lacking. The aim of this study was to measure VTE risk and associated mortality in general surgery using linked primary care and hospital databases, to improve our understanding of harm from VTE that arises beyond hospital stay.

**Methods:**

This was a longitudinal cohort study using nationally linked primary care (Clinical Practice Research Datalink, CPRD), hospital administrative (Hospital Episodes Statistics, HES), population statistics (Office of National Statistics, ONS) and National Cancer Intelligence Network databases. Routinely collected information was used to quantify 90-day in-hospital VTE, 90-day post-discharge VTE and 90-day mortality in adults undergoing one of twelve general surgical procedures between 1st April 1997 and 31st March 2012. The earliest postoperative recording of deep vein thrombosis or pulmonary embolism in CPRD, HES and ONS was counted in each patient. Covariates from multiple datasets were combined to derive detailed prediction models for VTE and mortality. Limitation included the capture of VTE presenting to healthcare only and the lack of information on adherence to pharmacological thromboprophylaxis as there was no data linkage to hospital pharmacy records.

**Results:**

There were 981 VTE events captured within 90 days of surgery in 168005 procedures (23.7/1000 patient-years). Overall, primary care data increased the detection of postoperative VTE by a factor of 1.38 (981/710) when compared with using HES and ONS only. Total VTE rates ranged between 3.2/1000 patient-years in haemorrhoidectomy to 118.3/1000 patient-years in esophagogastric resection. Predictors of VTE included emergency surgery (OR = 1.91 95%CI 1.60–2.28, p<0.001), age (OR = 1.02 95%CI 1.02–1.03, p<0.001), body mass index (OR = 1.03 95%CI 1.01–1.04, p<0.001), previous VTE (OR = 8.07 95%CI 6.61–9.83, p<0.001), length of stay (OR = 1.00 95%CI 1.00–1.00, p = 0.007) and cancer stages II (OR = 1.38 95%CI 1.03–1.87, p = 0.033), III (OR = 1.50 95%CI 1.11–2.01, p = 0.008) and IV (OR = 1.63 95%CI 1.03–2.59, p = 0.038). Major organ resections had the greatest odds of VTE when adjusted for other risk factors including length of hospital stay. Post-discharge VTE accounted for 64.8% (636/981) of all recorded VTE. In-hospital VTE (165.4/1000 patient-years) was recorded more frequently than post-discharge VTE (16.2/1000 patient-years). Both in-hospital (OR = 2.07 95%CI 1.51–2.85, p<0.001) and post-discharge (OR = 4.03 95%CI 2.95–5.51, p<0.001) VTE independently predicted 90-day mortality. In patients who died and VTE was recorded on HES or CPRD (n = 56), VTE was one of the causes of death in 37.5% (21/56) of cases.

**Conclusions:**

A large proportion of postoperative VTE was detected in primary care. Evaluation of linked databases was a useful way of measuring postoperative VTE at population level. These resources identified a significant association between post-discharge VTE and mortality in general surgery.

## Introduction

Postoperative venous thromboembolism (VTE) can have devastating effects on patients. However, VTE rates are difficult to estimate at population-level. In surgery, increasing trends towards day case operating, enhanced recovery and early discharge mean that a significant proportion of VTE may present after hospital discharge and be treated in the ambulatory setting.[1, 2]Previous studies report that VTE risk persists up to 90 days after surgery and beyond hospital stay.[3, 4] The National Institute for Health and Care Excellence (NICE) suggest the need to scrutinize primary care data to gain a complete picture of postoperative VTE at a population level and shape future thromboprophylaxis policy.[5] Primary care physicians in the United Kingdom (UK) are well placed to observe post-discharge adverse events as they are often the first point of contact for patients in this scenario.[6]

Studies evaluating Hospital Episodes Statistics (HES), an administrative hospital database in England, have estimated postoperative VTE rates in some surgical procedures.[7–9] However, HES by itself is limited to capturing in-hospital adverse events and those recorded during readmission. Linkage to primary care electronic health records allows for a multisource perspective of patients’ entire health journeys. Linked databases offer patient-level information by combining hospital administrative, primary care, population statistics and cancer registry databases. These resources have previously been used to quantify the risk of post-discharge VTE in patients with colorectal cancer and in hospitalized pregnant women.[10, 11] Whether linked data can evaluate postoperative VTE rates across different surgical procedures in patients with varying degrees of VTE risk has not previously been assessed.

Thromboprophylaxis guidelines in the UK have originated from the THRIFT (Thromboembolic Risk Factors Consensus Group) recommendations, which were derived from in-patient data.[12] More recently, the importance of outpatient thromboprophylaxis to prevent post-discharge VTE in major cancer resection has been recognized.[13] The current NICE guidelines recommend that general surgical patients receive either no thromboprophylaxis, in-patient thromboprophylaxis or thromboprophylaxis to 28 days after surgery based on defined VTE risk criteria.[5] Analysis of linked hospital and primary care data may improve our understanding of post-discharge VTE risk and help develop future policy for outpatient thromboprophylaxis.

This study aims to measure postoperative VTE and associated mortality up to 90 days following general surgical procedures using linked hospital and primary care databases in England. The study evaluates surgical VTE risk, particularly that which continues in the outpatient setting, to explore whether these resources can increase our knowledge about surgical harm from a primary care perspective.

## Methods

### Database Linkage

The UK has one of the largest numbers of electronic primary care health records in the world that can help evaluate the quality of care provided by the National Health Service (NHS).[14] The Clinical Practice Research Datalink (CPRD), formerly the General Practice Research Database, has generated the highest number of peer-reviewed publications from primary care data of any such database globally.[15] Other databases in the UK include The Health Improvement Network, Secure Anonymized Information Linkage System and Egerton Medical Information System, which offer fewer patients with linkage to other databases compared with CPRD. In CPRD, over 600 primary care practices covering 8.5% of the UK population are registered to use the Vision software for recording information during consultations. Anonymized patient information is automatically uploaded to CPRD with an ‘opt-out’ clause for patients in consenting practices. About 50% of CPRD practices are linked to in-patient HES data, which contain procedural and diagnostic information for each episode of hospital care in England. Additionally, 95% of HES-linked practices also have Office of National Statistics (ONS) linkage to death certificates that contain codes for causes of death. Only practices with HES and ONS linkage were included in the study. CPRD also offer linkages to clinical registries. Out of these, the National Cancer Intelligence Network (NCIN) database was relevant to this study. In patients with CPRD, HES and ONS linkage, 90% of cancer patients were linked to NCIN. Patient linkage was performed centrally using the NHS number.[16] Database coverage was for index procedures performed between 1^st^ April 1997 and 31^st^ March 2012 with at least 90 days follow-up (to 30^th^ June 2012) in all databases.

### Study Population

Surgical patients and indications for surgery were identified from the HES database as the accuracy of operative and diagnostic coding has previously been validated.[17] Patients undergoing one of twelve index general surgical procedures coded in HES using the Classification of Interventions and Procedures from the Office of Population Census and Surveys (OPCS) version 4.4 and the International Classification of Diseases version 10 (ICD-10) (S1 Table) were included. These procedures represent the majority of emergency and elective general surgical operations in UK hospitals. Patients were included in the analysis if they were coded for an index procedure by a primary (in the first position of an episode of care) OPCS code for the first chronological operation during hospital admission. Only those patients in whom the index episode also specified a designated diagnosis (coded in the first position for diagnostic code by ICD-10) as the indication for surgery were included in the study. Only adults aged 18 or over were eligible. Patients were included if they met the up-to-standard criteria for data quality set by CPRD. These criteria incorporated patient and practice level parameters that filtered out poor quality data by identifying discrepancies in coding and periods of missing information.

### Venous Thromboembolism Rate

VTE events were counted to calculate 30, 60 and 90-day VTE rates by combining data from CPRD, HES and ONS using diagnostic codes for deep vein thrombosis (DVT) and pulmonary embolism (PE) (S2 Table). In CPRD, medcodes are mapped equivalents of the widely used READ clinical coding system in primary care in the UK. Codes for VTE in CPRD have been reported previously.[18] Validation against hospital correspondence and physician questionnaires found them to be 84% accurate.[19] In CPRD, VTE was defined as a code for DVT or PE with evidence of anticoagulation between 15 days before and 90 days after the event, or death within 30 days of the event as previously described.[11, 19, 20] ICD-10 codes were used to identify DVT and PE in HES and ONS. In HES, VTE was recorded if it was coded during the index hospital admission (any diagnostic code except for the diagnostic code in the first position of the index episode) or if it was the reason for hospital readmission (first diagnostic code in an episode of readmission) within 90 days of surgery. In ONS, a VTE event was counted if it was recorded in a death certificate and death occurred within 90 days of surgery. Only the earliest recording of VTE after surgery in any database was counted in each patient. Short-term VTE rates were calculated for the most commonly performed procedures to allow for comparisons with studies that report VTE rates but have shorter follow-up.

### Venous Thrombolism Risk

Multiple regression analysis for postoperative VTE was performed by entering risk factors outlined in the NICE thromboprophylaxis guidelines.[5] In addition, cancer stage was incorporated into the model in light of growing recognition of cancer-specific variables in predicting surgical outcome.[21] Year of surgery was also included to assess whether there were any associations over time. The covariates used to evaluate VTE risk together with the corresponding databases from which they were derived from are listed below:

1. age (all databases)
2. gender (all databases)
3. previous VTE (CPRD and HES)
4. body mass index (BMI) (CPRD)
5. comorbidity (HES)
6. emergency surgery (HES)
7. length of stay (HES)
8. year of surgery (HES)
9. index surgical procedure (HES)
10. cancer stage (NCIN)
11. oral contraceptive pill (OCP) (CPRD)
12. hormone replacement therapy (HRT) (CPRD)

Cancer stage was categorized according to the TNM classification system with stage grouping I-IV.[22] Previous VTE was determined by identifying VTE codes in HES and CPRD before surgery. ICD-10 codes for fifteen different comorbidities (excluding malignancy and metastases) were adopted from a previous study.[23] The presence of one or more comorbidities was evaluated against VTE risk in accordance with the risk assessment method described in the guidelines. The use of OCP and HRT were defined as a prescription for British National Formulary codes 7.3.1, 7.3.2, 13.6.2 and 6.4.1.1 within three months before surgery, as previously described.[18]

### In-Hospital and Post-Discharge Venous Thromboembolism

Whether VTE was recorded before or after hospital discharge was determined from the discharge date of the index hospital admission in HES. Post-discharge VTE was defined as those recorded in CPRD or ONS after the discharge date of the index admission, or those leading to hospital readmission after this date in HES. The remaining VTE events recorded in the index admission in HES or before the index discharge date in CPRD and ONS were classified as in-hospital VTE.

### Mortality

Death, date of death and cause of death were defined from ONS data. Multiple regression analysis explored the risk-adjusted effects of in-hospital and post-discharge VTE on mortality separately. To assess whether VTE recorded in CPRD and HES contributed to death, death certificates were evaluated after excluding patients with VTE recorded in ONS data only. VTE-related death was defined as death with recorded VTE in either CPRD or HES databases and VTE recorded as one of the causes of death. As VTE was always recorded before or at the time of death in this context, VTE was considered to cause or contribute to death these patients.

### Statistical Analysis

SPSS version 22.0 (SPSS for Windows, Chicago, Illinois) was used for all analyses. For logistic regression analysis all variables were entered into the model from the outset. Age, length of stay and BMI were treated as continuous variables. All other variables were categorical. For the analysis of VTE risk, gender and hormone treatment were combined in a single categorical variable. A p<0.05 was statistically significant for all tests.

### Missing Data

Only recordings within five years before surgery were used to estimate preoperative BMI. Only BMI recordings of 12–75 were considered valid to exclude out-lying values. Multiple imputation of missing values modeled BMI against age, sex, socioeconomic status and diabetes. Missing data from the NCIN database were handled by categorizing patients as unknown cancer stage.

## Results

Data from 353 practices with patient-level linkage of CPRD, HES, ONS and NCIN were studied. From 1^st^ April 1997 to 31^st^ March 2012, 168005 primary surgical procedures were evaluated in 159039 patients. Patient characteristics and mortality for the twelve different procedures are presented in Table 1. The mean time from preoperative recording of BMI to surgery was 1.4 years. The proportion of patients with recorded BMI within five years before surgery ranged from 55.1% to 71.4%, except for in bariatric surgery where 95.1% of patients had these data. Previous history of VTE was recorded in 1.32% (2222/168005) of patients. Cancer stage data were available in 58.5% (23645/40452) of patients with malignancy. The availability of cancer stage data was highest (80.1%) in colorectal cancer resection (11629/14521) and lowest (9.5%) in small bowel resection (19/200).

**Table 1:** Characteristics and 90-day mortality for patients undergoing general surgical procedures between 1 April 1997 and 31 March 2012.

	Patients	Malignancy	Age	Gender	BMI	Mortality
Procedure						
	n	n (%)	Median (IQR)	female (%)	mean	n (%)
Antireflux surgery	1219	0 (0)	47 (38–57)	545 (44.7)	27.8	2 (0.16)
Appendicectomy	13216	0 (0)	36 (24–49)	6099 (46.1)	26.5	42 (0.32)
Bariatric surgery	1475	0 (0)	44 (37–52)	1148 (77.8)	48.1	4 (0.27)
Breast excision	21383	21383 (100)	61 (51–70)	21383 (100)	27.2	100 (0.47)
Cholecystectomy	36197	0 (0)	53 (40–65)	27330 (75.5)	28.9	139 (0.38)
Colorectal resection	18923	14521 (76.7)	69 (59–77)	8964 (47.4)	26.6	1360 (7.19)
Haemorrhoidectomy	17554	0 (0)	52 (42–64)	8190 (46.7)	26.9	37 (0.21)
Hepatopancreatobiliary resection	1486	1486 (100)	65 (57–72)	650 (43.7)	26.7	80 (5.38)
Inguinal hernia repair	46303	0 (0)	62 (49–72)	3357 (7.3)	25.8	222 (0.48)
Esophagogastric resection	2091	2091 (100)	68 (59–74)	599 (28.6)	26.4	171 (8.18)
Small bowel resection	1727	200 (11.6)	65 (48–76)	990 (57.3)	25.6	229 (13.3)
Thyroid/parathyroid excision	6431	771 (12.0)	52 (40–64)	5205 (80.9)	27.0	40 (0.62)
Total	168005	40452 (24.1)	58 (44–69)	84460 (50.27)	27.4	2426 (1.44)

IQR = Interquartile range

BMI = body mass index

### Venous Thromboembolism Rate

**Fig 1:**
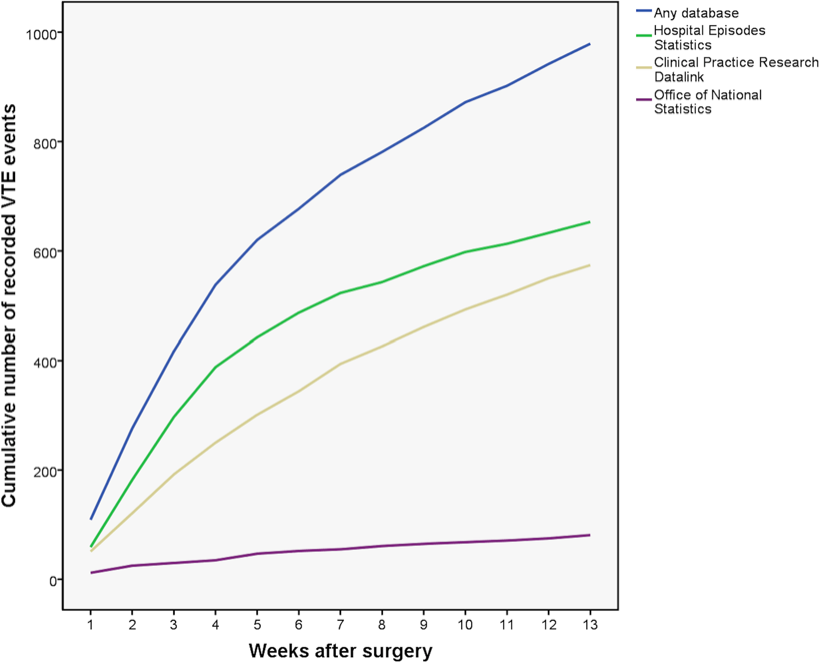
Cumulative postoperative VTE according to recorded database in general surgical procedures performed between 1 April 1997 and 31 March 2012.

**Fig 2:**
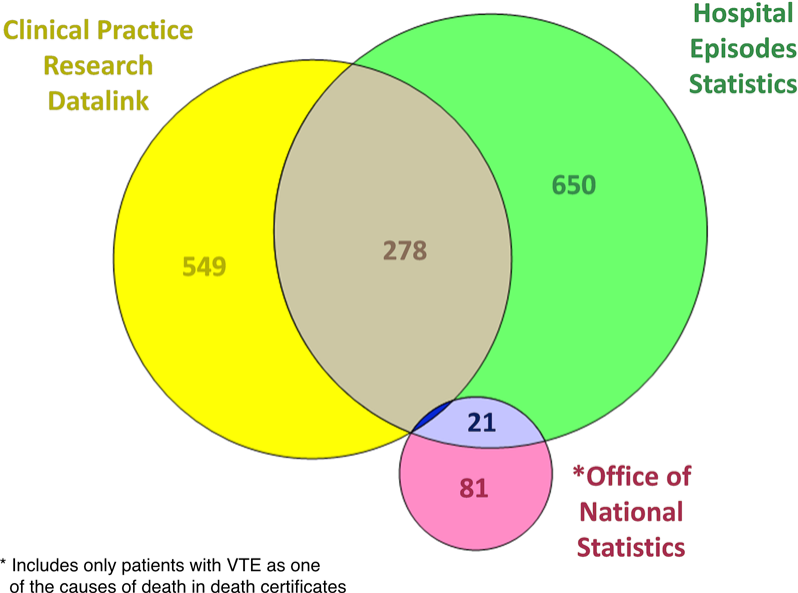
Venn diagram of VTE events according to recorded database showing overlap between databases in general surgical procedures performed between 1 April 1997 and 31 March 2012.

Different datasets recorded VTE at different rates over time (Fig 1). Overall, CPRD recorded 56.0% (549/981), HES recorded 66.3% (650/981) and ONS recorded 8.3% (81/981) of all recorded VTE events. VTE was recorded in CPRD alone and not in any other database in 23.8% (136/571), 25.6% (207/810) and 27.6% (271/981) of VTE at 30, 60 and 90 days respectively. Some VTE events were recorded in more than one database (Fig 2). When VTE was recorded in two databases in the same patient, the overlap between CPRD and HES was 28.3% (278/981) and HES and ONS was 2.1% (21/981). Compared with using HES and ONS without CPRD, primary care data increased the detection of postoperative VTE by a factor of 1.38 (981/710).

When combining information from different databases, postoperative VTE was recorded in 571 (0.34%), 810 (0.48%) and 981 (0.58%) procedures at 30, 60 and 90 days respectively. Thirty-day VTE rates for the four most commonly performed procedures were 1.26% (238/18923), 0.22% (47/21383), 0.13% (60/46303) and 0.17% (56/32164) for colorectal resection, breast excision, inguinal hernia repair and laparoscopic cholecystectomy respectively. Ninety-day VTE rates ranged from 0.08% (14/17554) following haemorrhoidectomy to 2.92% (61/2091) following esophagogastric resection (Table 2). PE was recorded more frequently than DVT in all three databases. CPRD recorded the largest proportion of DVTs. There were 285, 398 and 75 PEs, and 283, 255 and 34 DVTs recorded in CPRD, HES and ONS respectively. Only in 5.6% (55/981) of procedures were both DVT and PE recorded in the same patient. Of these, DVT was recorded chronologically before PE in 56.4%(31/55) of patients while DVT was recorded on the same day as PE in 14.5% (8/55) of patients.

**Table 2:** VTE captured at 30, 60 and 90 days according to recorded database in general surgical procedures performed between 1 April 1997 and 31 March 2012.

Procedure	Number of patients	30 day	30 day	30 day	60 day	60 day	60 day	90 day	90 day	90 day	Overall rate n (%)
		CPRD	HES	ONS	CPRD	HES	ONS	CPRD	HES	ONS	
Antireflux surgery	1219	4	1	0	1	3	0	0	0	0	7 (0.57)
Appendicectomy	13216	27	37	*	14	7	0	4	1	0	65 (0.49)
Bariatric surgery	1475	2	1	0	1	1	0	0	0	0	4 (0.27)
Breast excision	21383	28	27	*	29	16	*	25	16	*	118 (0.55)
Cholecystectomy	36197	31	56	*	13	15	0	17	7	0	109 (0.30)
Colorectal resection	18923	90	182	19	69	65	14	46	38	6	400 (2.11)
Haemorrhoidectomy	17554	3	3	*	3	2	0	0	2	*	14 (0.08)
Hepatopancreatobiliary resection	1486	5	10	*	6	4	0	6	8	*	31 (2.09)
Inguinal hernia repair	46303	35	39	*	21	16	*	17	9	*	112 (0.24)
Esophagogastric resection	2091	9	27	*	9	6	*	5	5	*	61 (2.92)
Small bowel resection	1727	7	20	*	8	11	*	3	5	0	44 (2.55)
Thyroid/parathyroid excision	6431	9	9	0	0	0	0	2	1	*	16 (0.25)
Total	168005	250	412	36	174	146	28	125	92	17	981 (0.58)

CPRD = Clinical Practice Research Datalink

HES = Hospital Episodes Statistics

ONS = Office of National Statistics

The overall rate of VTE captured by all databases exceeds the sum of number of VTE events in each database due to overlap of VTE recording between databases

* Restricted disclosure in accordance with the Health and Social Care Information Centre small numbers policy

### Venous Thromboembolism Risk

Regression analysis identified emergency surgery (OR = 1.91 95% CI 1.60–2.28, p<0.001), age (OR = 1.02 95%CI 1.02–1.03, p<0.001), BMI (OR = 1.03 95%CI 1.01–1.04, p = 0.001), previous history of VTE (OR = 8.07 95%CI 6.61–9.83, p<0.001), length of hospital stay (OR = 1.00 95%CI 1.00–1.00, p = 0.007), stage II cancer (OR = 1.38 95%CI 1.03–1.87, p = 0.033), stage III cancer (OR = 1.50 95%CI 1.11–2.01, p = 0.008) and stage IV cancer (OR = 1.63 95%CI 1.03–2.59, p = 0.038) as independent predictors of postoperative VTE (Table 3). Major organ resections (colorectal resection, small bowel resection, hepatopancreatobiliary resection, esophagogastric resection) were associated with the highest odds for VTE when adjusted for other covariates. While BMI was an independent risk factor for VTE, bariatric surgery per se was associated with a low risk of VTE when adjusted for predictor covariates including BMI.

**Table 3:** Multiple logistic regression analysis for 90-day VTE for patients undergoing general surgical procedures between 1 April 1997 and 31 March 2012.

Dependent factor = 90-day VTE	Odds ratio (95% CI)	Significance
Emergency surgery	1.91 (1.60–2.28)	p<0.001
Age	1.02 (1.02–1.03)	p<0.001
BMI	1.03 (1.01–1.04)	p = 0.001
Comorbidity	1.16 (1.00–1.35)	p = 0.051
Year of surgery	0.99 (0.97–1.00)	p = 0.127
Previous VTE	8.07 (6.61–9.83)	p<0.001
Length of stay	1.00 (1.00–1.00)	p = 0.007
*SURGICAL PROCEDURE*		
Haemorrhoidectomy	*Reference*	
Bariatric surgery	2.32 (0.73–7.38)	p = 0.156
Inguinal hernia repair	2.73 (1.56–4.77)	p<0.001
Thyroid /parathyroid excision	3.14 (1.57–6.27)	p = 0.002
Cholecystectomy	3.41 (1.95–5.96)	p<0.001
Breast excision	4.39 (2.37–8.11)	p<0.001
Appendicectomy	4.80 (3.53–6.52)	p<0.001
Antireflux surgery	8.38 (5.26–13.35)	p<0.001
Colorectal resection	11.28 (6.31–20.20)	p<0.001
Small bowel resection	12.81 (6.85–24.00)	p<0.001
Hepatopancreatobiliary resection	15.03 (7.37–30.64)	p<0.001
Esophagogastric resection	17.24 (9.03–32.94)	p<0.001
*CANCER STAGE*		
No cancer	*Reference*	
Cancer stage I	1.26 (0.89–1.79)	p = 0.200
Cancer stage II	1.38 (1.03–1.87)	p = 0.033
Cancer stage III	1.50 (1.11–2.01)	p = 0.008
Cancer stage IV	1.63 (1.03–2.59)	p = 0.038
Cancer stage unknown	1.33 (0.99–1.79)	p = 0.060
*GENDER AND HORMONE THERAPY*		
Women < 45 years no OCP	*Reference*	
Women <45 years with OCP	1.86 (0.90–3.84)	p = 0.094
Women >45 years no HRT	1.48 (1.03–2.13)	p = 0.035
Women >45 years with HRT	1.48 (0.86–2.55)	p = 0.158
All men	1.44 (1.00–2.06)	p = 0.048

BMI = body mass index

VTE = venous thromboembolism

OCP = oral contraceptive pill

HRT = hormone replacement therapy

### In-Hospital and Post-Discharge Venous Thromboembolism

In-hospital VTE accounted for 35.2% (345/981) and post-discharge VTE accounted for 64.8% (636/981) of all recorded VTE (Table 4). The proportion of post-discharge VTE out of all VTE events ranged from 31.8% to 88.4% according to surgical procedure. HES recorded 86.4% (298/345) of all in-hospital VTE (VTE recorded during the index hospital admission) while CPRD recorded 71.7% (456/636) of all post-discharge VTE (recorded after the discharge date). The overall VTE rate was 23.7 per 1000 patient years. When taking into account the length of hospital stay in each patient, the frequency of in-hospital VTE was 165.4 per 1000 patient years while the frequency of post-discharge VTE was 16.2 per 1000 patient years.

**Table 4:** Length of hospital stay and rate of in-hospital and post-discharge VTE for patients undergoing general surgical procedures between 1 April 1997 and 31 March 2012.

Procedure	Median length of stay (IQR)	Total VTE	Total VTE	In-hospital VTE	In-hospital VTE	In-hospital VTE	Post-discharge VTE	Post-discharge VTE	Post-discharge VTE
		Number of events	Rate per 1000 patient years	Number of events	Rate per 1000 patient years	Proportion of VTE %	Number of events	Rate per 1000 patient years	Proportion of VTE %
Antireflux surgery	2 (1–4)	7	23.3	1	91.9	14.3	6	20.7	85.7
Appendicectomy	3 (2–5)	65	19.9	24	154.1	36.9	41	13.2	63.1
Bariatric surgery	2 (1–4)	4	11.0	1	76.4	25.0	3	8.6	75.0
Breast excision	3 (1–5)	118	22.4	17	77.6	14.4	101	20.0	85.6
Cholecystectomy	2 (1–3)	109	12.2	32	102.9	29.4	77	8.9	70.6
Colorectal resection	12 (8–18)	400	85.7	178	207.9	44.5	222	58.3	55.5
Haemorrhoidectomy	0 (0–1)	14	3.2	3	85.2	21.4	11	2.6	78.6
Hepatopancreatobiliary resection	11 (8–18)	31	84.6	11	168.0	35.5	20	66.5	64.5
Inguinal hernia repair	0 (0–1)	112	9.8	13	90.5	11.6	99	8.8	88.4
Esophagogastric resection	15 (11–21)	61	118.3	28	246.6	45.9	33	82.1	54.1
Small bowel resection	14 (9–24)	44	103.3	30	288.9	68.2	14	43.5	31.8
Thyroid/parathyroid excision	2 (1–4)	16	10.1	7	119.7	43.8	9	5.9	56.3
Total	2 (0–5)	981	23.7	345	165.4	35.2	636	16.2	64.8

IQR = interquartile range

VTE = venous thromboembolism

### Mortality

There were 2426 deaths up to 90 days after surgery irrespective of whether VTE was the cause of death. Mortality in patients with recorded VTE was 11.8% (116/981). Over half of the deaths in patients with recorded VTE occurred after hospital discharge (51.7% — 60/116). Regression analysis revealed that both in-hospital (OR = 2.07 95%CI 1.51–2.85, p<0.001) and post-discharge VTE (OR = 4.03 95%CI 2.95–5.51, p<0.001) were independent predictors of mortality. Post-discharge VTE was associated with higher odds of death than in-hospital VTE. For the remaining covariates, emergency surgery, male gender, length of stay and cancer stage IV independently predicted 90-day mortality (Table 5). In those patients who died and in whom a VTE event was recorded on HES or CPRD (n = 56), VTE was recorded as one of the causes of death in 37.5% (21/56) of patients. In these VTE-related deaths, death was at least partly caused by VTE.

**Table 5:** Multiple logistic regression analysis for 90-day mortality for patients undergoing general surgical procedures between 1 April 1997 and 31 March 2012.

Dependent factor = 90-day mortality	Odds ratio (95% CI)	Significance
		
Emergency surgery	5.13 (4.62–5.70)	p<0.001
Male gender	1.07 (1.07–1.08)	p<0.001
Age	0.92 (0.84–1.01)	p = 0.094
BMI	0.99 (0.98–1.00)	p = 0.003
Comorbidity	0.99 (0.99–1.00)	p<0.001
Length of stay	2.48 (2.27–2.71)	p<0.001
*SURGICAL PROCEDURE*		
Haemorrhoidectomy	*Reference*	
Bariatric surgery	4.07 (1.42–11.65)	p = 0.009
Inguinal hernia repair	1.22 (0.86–1.74)	p = 0.269
Thyroid /parathyroid excision	3.20 (2.04–5.03)	p<0.001
Cholecystectomy	1.47 (1.03–2.10)	p = 0.041
Breast excision	1.78 (1.18–2.68)	p = 0.006
Appendicectomy	0.84 (0.54–1.32)	p = 0.454
Antireflux surgery	1.56 (0.75–3.24)	p = 0.540
Colorectal resection	9.95 (7.00–14.14)	p<0.001
Small bowel resection	10.63 (7.34–15.39)	p<0.001
Hepatopancreatobiliary resection	10.93 (7.03–16.99)	p<0.001
Esophagogastric resection	18.22 (12.23–27.15)	p<0.001
*CANCER STAGE*		
No cancer	*Reference*	
Cancer stage I	0.89 (0.71–1.13)	p = 0.337
Cancer stage II	0.89 (0.75–1.06)	p = 0.197
Cancer stage III	1.12 (0.94–1.32)	p = 0.208
Cancer stage IV	1.99 (1.74–2.27)	p<0.001
Cancer stage unknown	1.21 (1.02–1.44)	p = 0.027
*VTE TYPE*		
No VTE	*Reference*	
In-hospital VTE	2.07 (1.51–2.85)	p<0.001
Post-discharge VTE	4.03 (2.95–5.51)	p<0.001

BMI = body mass index

VTE = venous thromboembolism

## Discussion

### Principal Findings of Study

Patient-level linkage to CPRD helped identify more VTE events compared with using HES and ONS only. CPRD increased detection of VTE by almost 40%. Combining databases helped reveal similar VTE rates as reported from the National Surgical Quality Improvement Program (NSQIP), a dedicated registry for surgical adverse events.[2, 24–26] The highest rates were observed amongst in-patients (76.4 to 288.9 per 1000 patient years) but VTE was also detected after hospital discharge (2.6 to 82.1 per1000 patient years). As there was little overlap between HES and CPRD, VTE is either being treated entirely in the outpatient setting or not being recorded by hospitals and subsequently captured in primary care. Predictors of VTE included patient factors such as age, BMI, history of VTE and moderate to advanced cancer stage, as well as surgical factors such as emergency surgery and prolonged length of stay. Post-discharge VTE was associated with significant increases in mortality (OR = 4.03) when modeled against other predictors of death. Although inferences on causality cannot be made, a high proportion of VTE were also recorded in death certificates when recorded in HES and CPRD. Overall, the present analysis revealed significant harm from VTE in general surgery identified in the primary care setting, highlighting areas where refinement in policy may benefit patients.

### Strengths and Weaknesses of Study

Surgical VTE rates were calculated using routinely collected population data, which requires less resources and allows for longer durations of follow-up compared with prospective national registries.[27] Follow-up in NSQIP is performed through case notes review and limited to 30 days after surgery. Combining routine databases may be a reliable way of gaining insights into population-level outcomes and complimenting the results from prospective analyses. The inclusion codes and definition of VTE used in this study were specific.[19, 20] Such a narrow definition was chosen as this methodology has been previously validated but this may lead to undercounting of VTE events. On HES data there is no present on arrival flag. Therefore, when evaluating patients readmitted to hospital, only a VTE code in the primary diagnosis category was included. VTE codes for PE and DVT were included, while codes for superficial phlebitis and portal vein thrombosis were excluded.[4, 18] VTE derived from CPRD was considered valid if there was evidence of anticoagulation.[19] For the purposes of risk stratification, linked databases offered high numbers of patients and covariates that helped develop a detailed model for VTE risk. Through the provision of highly granular information on a large scale, linked routine data may serve as the foundation for individualizing prophylaxis strategies in the future.

This study identified harm that presented to healthcare. The derived incidence of VTE was therefore lower than in prospective studies with clinical follow-up or assessment by imaging.[28] As harm presenting to healthcare may vary considerably, data-driven research must take into account the type of health system from which the data is being generated. Another limitation of the study is the lack of information on pharmacological thromboprophylaxis making direct comparison of prescription data with CPRD challenging. Hospital pharmacy data were not present on HES, therefore it was not possible to determine adherence to thromboprophylaxis guidelines. Further linkage to hospital pharmacy records would clarify whether guidelines are being followed.

### Comparison with Previous Studies

Population-based studies using HES and ONS suggested that the incidence of VTE is increased up to 90 days after in-patient surgery (31.2 per 1000 patient years) and day-case surgery (4.8 per 1000 patient years).[4] Higher rates were observed in our study through the addition of primary care data. A significant number (51.7%) of deaths with a recording of VTE in at least one database occurred after hospital discharge. Lester and colleagues evaluated hospital admissions using HES and ONS and found that 37.6% (625/1661) of VTE-related deaths were post-discharge.[29] The higher rate in the present study highlights the importance of this mechanism of harm in general surgery. Other authors have used linked hospital and primary care data to derive disease-specific VTE rates in the primary care population.[11, 30, 31] Walker et al reported incidences of 13.9 per 1000 patient years in cancer and 3.0 per 1000 patient years in non-cancer populations.[20] The higher VTE rates in our study reflect the impact of surgical intervention on patients. In another study using the same linked dataset, the postoperative VTE rate for 4963 colorectal cancer resections was found to be 45.1 per 1000 patient years.[11] The lower rate in this study compared with our results may have arisen from differences in cohort selection and analytical methods. In our study, all HES-recorded VTE were included regardless of whether patients had prescription data for anticoagulation. There may also have been differences in codes used to identify VTE. Codes used in previous studies were not published so it was not possible to determine whether they were the same as in our study.[11, 20]

In terms of routinely collected data from abroad, hospital administrative data in the United States have demonstrated VTE risk persisting up to 90 days after surgery.[3] One study reports VTE rates of 1.1%, 1.5%, 0.4%, 0.2%, 0.2% and 0.1% for colorectal resection, small bowel resection, inguinal hernia repair, appendicectomy, laparoscopic cholecystectomy and thyroid/parathyroid resection respectively, generally lower than in the current study. The proportion of post-discharge VTE from these data range between 40–75%. Using prospectively collected NSQIP data, 30-day VTE rates are reported to be 1.90%, 0.23%, 0.09% and 0.19% in colorectal resection, breast excision, hernia repair and laparoscopic cholecystectomy respectively, similar to our study.[2, 24–26] Post-discharge VTE only accounts for about a third of VTE in the NSQIP database, because the follow-up is limited to 30 days.[1, 2] While the proportion of DVT and PE is not reported from administrative data, NSQIP reveals that about two-thirds of postoperative VTE is accounted for by DVT. The lower proportion of DVT in our study reflects the difference in methods of follow-up. While in NSQIP, patients are actively contacted during prospective follow-up to be questioned about symptoms of complications, our study relies on patients to present to healthcare. Our finding suggests that PE is more likely to present to healthcare than DVT. In linked database analysis, primary care data increased the detection of DVT, which were captured less reliably in hospital administrative and death certificate data.

### Implications for Policy and Future Direction

The identification of deaths related to post-discharge VTE reiterates the importance of thromboprophylaxis and early intervention in preventing harm from VTE.[32] Using the same dataset, Sultan et al. recommended extended thromboprophylaxis in pregnant women discharged from hospital.[10] Walker et al. also proposed that thromboprophylaxis beyond 28 days may benefit patients with advanced colorectal cancer receiving multimodal treatment.[11] In the year 2010, NICE introduced recommendations for extended thromboprophylaxis to 28 days after surgery in patients undergoing major cancer resection. Our analysis reveals that other procedures may also be associated with substantial post-discharge VTE risk. Some of these patients may benefit from extended thromboprophylaxis in addition to thromboprophylaxis during in-hospital stay. In addition to drives to shorten hospital stay, increasing complexity of treatments and changing population demographics may be some of the factors contributing to higher post-discharge VTE risk associated with general surgical procedures than previously appreciated. Prospective studies and randomized trials remain as gold standards to evaluate whether further refinement in policy could benefit some patient groups. Linked databases can improve our knowledge at population level serving as a platform for economic analysis and evaluation of safety. It would be important to consider both the impact on mortality and the health costs of VTE incurred to surviving patients.[33]

Other adverse events may also be suited for measurement in primary care. This may widen the scope of using these resources to monitor patient safety and healthcare performance. In light of drives to transfer clinical skills and resources from hospitals to primary care, service providers and commissioners should embrace the use of linked routine databases as a valuable way of evaluating process and outcome within integrated health systems.[34] Further work is necessary to validate these resources through direct comparisons with other types of data. As the linkage of healthcare databases increases, methods for coding and reporting of harm in multiple datasets should be standardized. CPRD is generated from one of several software systems used in primary care. As 98% of practices in the UK use electronic health records, achieving uniformity of primary care software systems and further population linkage with hospital records may allow for these resources to be developed for the purposes of service evaluation and driving quality improvements on a national scale. Furthermore, the identification of significant levels of post-discharge harm may be relevant to health systems with less integration of primary and secondary care services. The presented findings highlight the need to bridge care and for hospitals to offer support when patients return to the community for recuperation after surgery.

### Conclusion

A large proportion of postoperative VTE was detected in the primary care setting. Evaluation of linked databases was a useful way of measuring postoperative VTE at population level. These resources identified a significant association between post-discharge VTE and mortality in general surgery.

## Supporting Information

S1 Table Surgical operations defined by procedural (OPCS) and diagnostic (ICD-10) codes in HES. (DOCX)

S2 Table Diagnostic codes for VTE in CPRD, HES and ONS. (DOCX)
