# Supplementary material for: Risk of Post-Discharge Venous Thromboembolism and Associated Mortality in General Surgery: A Population-Based Cohort Study Using Linked Hospital and Primary Care Data in England
Source: PLoS One. 2015 Dec 29;10(12):e0145759. doi: 10.1371/journal.pone.0145759 (PMC4694702; doi:10.1371/journal.pone.0145759)
Supplement: S1 Table — (DOCX) [file pone.0145759.s001.docx]

| S Table 1 Surgical operations defined by procedural (OPCS) and diagnostic (ICD-10) codes in HES | |  |
| --- | --- | --- |
|  |  |  |
| **Surgical procedure** | **OPCS codes** | **ICD-10 codes** |
|  |  |  |
| Antireflux surgery | G23, G24 | K21 |
| Appendicectomy | H01, H02 | K35, K81, K82 |
| Bariatric surgery | G281, G282, G283,G284, G285, G288, G289, G301, G302, G303, G304, G308, G309, G311, G312, G313, G314, G315, G316, G318, G319, G310, G320, G321, G322, G323, G324, G325, G328, G329, G330, G331, G332, G333, G335, G336, G338, G339, G716 | E660, E661, E662, E668, E669 |
| Breast excision | B27, B28 | C50 |
| Cholecystectomy | J18 | K80, K81, K82 |
| Colorectal resection | H06, H07, H08, H09, H10, H335, H041, H043, H048, H049, H05, H11, H331, H332, H333, H334, H336, H337, H338, H339 | C18, C19, C20, C21, C26, K50, K51, K57 |
| Esophagogastric resection | G01, G02, G03, G27, G28 | C15, C16 |
| Haemorrhoidectomy | H51, H52, H53 | I84 |
| Hepatopancreatobiliary resection | J02, J031, J035, J18, J27, J55, J56, J57, J58 | C17, 18, C19, C20, C21, C22, C23, C24, C25, C26, C78 |
| Inguinal hernia repair | T20 | K40 |
| Small bowel resection | G58, G59, G69, G70 | C8, C17, K50, K55, K56, K57, K660 |
| Thyroid/parathyroid excision | B08, B12, B14, B16 | C73, C75, D34, D351, E01, E02, E03, E04, E05, E06, E750, E21 |
|  |  |  |
| OPCS = Office of Population Census and Surveys | |  |
| ICD-10 = International Classification of Diseases version 10 | |  |
