# Supplementary material for: Risk of Post-Discharge Venous Thromboembolism and Associated Mortality in General Surgery: A Population-Based Cohort Study Using Linked Hospital and Primary Care Data in England
Source: PLoS One. 2015 Dec 29;10(12):e0145759. doi: 10.1371/journal.pone.0145759 (PMC4694702; doi:10.1371/journal.pone.0145759)
Supplement: S2 Table — (DOCX) [file pone.0145759.s002.docx]

| S Table 2 Diagnostic codes for VTE in CPRD, HES and ONS | | |  |
| --- | --- | --- | --- |
|  |  |  |  |
| **Database** | **Coding system** | **Codes for DVT** | **Codes for PE** |
|  |  |  |  |
|  |  |  |  |
| CPRD | medcode | 1224, 824, 428, 3392, 9255, 22038, 25478, 3576, 32002, 42158, 3466 | 18121, 1266, 24444, 9701 |
|  |  |  |  |
| HES and ONS | ICD-10 | I801, I802 | I260, I269 |
|  |  |  |  |
|  |  |  |  |
| DVT = deep vein thrombosis | |  |  |
| PE = pulmonary embolism | |  |  |
| CPRD = Clinical Practice Research Datalink | | |  |
| HES = Hospital Episodes Statistics | |  |  |
| ONS = Office of National Statistics | |  |  |
| ICD-10 = International Classification of Diseases version 10 | | |  |
|  |  |  |  |
